# Epigenetic and Transcriptional Alterations in Human Adipose Tissue of Polycystic Ovary Syndrome

**DOI:** 10.1038/srep22883

**Published:** 2016-03-15

**Authors:** Milana Kokosar, Anna Benrick, Alexander Perfilyev, Romina Fornes, Emma Nilsson, Manuel Maliqueo, Carl Johan Behre, Antonina Sazonova, Claes Ohlsson, Charlotte Ling, Elisabet Stener-Victorin

**Affiliations:** 1 Department of Physiology, Institute of Neuroscience and Physiology, Sahlgrenska Academy, University of Gothenburg, Gothenburg, Sweden; 2 Epigenetics and Diabetes, Department of Clinical Sciences, Lund University Diabetes Centre, Lund University, Clinical Research Centre, Malmö, Sweden; 3 Department of Physiology and Pharmacology, Karolinska Institutet, Stockholm, Sweden; 4 Endocrinology and Metabolism Laboratory, Department of Medicine, West division, University of Chile, Santiago, Chile; 5 The Wallenberg Laboratory and Sahlgrenska Center for Cardiovascular and Metabolic Research, Institute of Medicine, Sahlgrenska Academy, University of Gothenburg, Gothenburg, Sweden; 6 Department of Obstetrics and Gynecology, Reproductive Medicine, Institute of Clinical Sciences, Sahlgrenska Academy, University of Gothenburg, Gothenburg, Sweden; 7 Department of Internal Medicine, Centre for Bone and Arthritis Research, Institute of Medicine, Sahlgrenska Academy, University of Gothenburg, Gothenburg, Sweden

## Abstract

Genetic and epigenetic factors may predispose women to polycystic ovary syndrome (PCOS), a common heritable disorder of unclear etiology. Here we investigated differences in genome-wide gene expression and DNA methylation in adipose tissue from 64 women with PCOS and 30 controls. In total, 1720 unique genes were differentially expressed (*Q* < 0.05). Six out of twenty selected genes with largest expression difference (*CYP1B1, GPT*), genes linked to PCOS (*RAB5B*) or type 2 diabetes (*PPARG, SVEP1*), and methylation (*DMAP1*) were replicated in a separate case-control study. In total, 63,213 sites (*P* < 0.05) and 440 sites (*Q* < 0.15) were differently methylated. Thirty differentially expressed genes had corresponding changes in 33 different DNA methylation sites. Moreover, a total number of 1913 pairs of differentially expressed “gene-CpG” probes were significantly correlated after correction for multiple testing and corresponded with 349 unique genes. In conclusion, we identified a large number of genes and pathways that are affected in adipose tissue from women with PCOS. We also identified specific DNA methylation pathways that may affect mRNA expression. Together, these novel findings show that women with PCOS have multiple transcriptional and epigenetic changes in adipose tissue that are relevant for development of the disease.

Polycystic ovary syndrome (PCOS) is the most common endocrine and metabolic disorder in women, with a worldwide prevalence of 7–15%^1^. The reproductive phenotype of PCOS includes hyperandrogenism, ovulatory disturbances, and polycystic ovarian morphology. The metabolic phenotype includes hyperinsulinemia with pancreatic β-cell dysfunction, insulin resistance, and obesity. These metabolic abnormalities often precede type 2 diabetes (T2D)^2^,^3^. Compensatory hyperinsulinemia stimulates androgen production and secretion by thecal cells and reduces the level of sex hormone binding globulin, which increases free androgens and further exacerbates PCOS symptoms^2^. Thus, there is a strong association between hyperinsulinemia and hyperandrogenemia that drives a specific vicious circle. Despite the detrimental effect of PCOS on women’s health, the etiology is not well understood. Genetic and environmental factors have been implicated in its development, but studies examining the impact of epigenetics on PCOS remain scarce^4^,^5^,^6^.

The considerable overlap between the reproductive and metabolic features of PCOS implicates adipose tissue dysfunction as a key feature of the disease^7^,^8^,^9^,^10^. Importantly, both lean and obese women with PCOS have aberrant adipose tissue morphology and function, as evidenced by enlarged subcutaneous adipocytes and decreased secretion of adiponectin—two factors that are strongly associated with insulin resistance^9^. Further, subcutaneous adipocytes from women with PCOS are resistant to insulin-stimulated glucose uptake and display inhibited lipolysis^11^,^12^. Moreover, altered adipose tissue expression of genes such as *PPARG*, *LEP*R, *TWIST1, CCL2* that may be important in PCOS pathophysiology have been identified^13^,^14^,^15^,^16^,^17^,^18^. These findings indicate that adipose tissue dysfunction may contribute to the pathogenesis of PCOS including insulin resistance and hyperandrogenism.

Emerging evidence from women with PCOS and from prenatally androgenized animal models suggests that maternal androgen excess predisposes offspring to PCOS^19^. An unfavorable intrauterine environment may lead to epigenetic changes that alter gene expression and increase risk of disease in adulthood^20^. Several lines of evidence support a role for epigenetic mechanisms in metabolic disturbances. For example, differential DNA-methylation in adipose tissue from subjects with T2D is associated with differential expression of genes involved in oxidative phosphorylation, mitochondrial function, and the metabolism of carbohydrates, amino acids, and lipids, all of which are relevant to the development of disease^21^. The possibility of an epigenetic component of PCOS was only recently explored^4^,^5^,^6^. Although hampered by minimal sample size^22^,^23^, these studies revealed differential DNA methylation and gene expression profiles in ovarian tissue within pathways related to the pathogenesis of PCOS. Also, epigenetic modification of *PPARGC1A* has been reported in granulosa cells from women with PCOS^24^.

Nevertheless, knowledge concerning the role of epigenetics in PCOS is limited. The genome-wide DNA methylation pattern has not been explored in human adipose tissue from women with and without PCOS, and genome-wide expression profiles have not been linked to the epigenome until just recently^25^. Therefore, our aim was to investigate genome-wide gene differences in expression and DNA methylation patterns in adipose tissue from 64 women with PCOS and 30 controls. In a separate case-control study, key findings were replicated in adipose tissue from 42 unrelated women.

## Results

### Clinical Characteristics

The characteristics of women in the two cohorts are presented in Table 1. In cohort 1, women with PCOS were older (30.0 ± 4.4 vs 27.6 ± 3.54) and had a higher BMI. (28.4 ± 7.1 vs 24.8 ± 5.0) Therefore, all analyzes in cohort 1 were adjusted for age and BMI including clinical characteristics, genome-wide gene expression and methylation. Women with PCOS had a higher HOMA-IR (homeostatic model assessment of insulin resistance) and higher levels of HbA1c and circulating testosterone than controls, and a lower glucose disposal rate (GDR) as measured by euglycemic hyperinsulinemic clamp. In cohort 2 replication was performed in cases and controls that were overweight or obese and matched for age (±5 years), weight (±4 kilogram), and body mass index (BMI) (±2) with controls. Women with PCOS in cohort 2 had higher HOMA-B (reflecting insulin secretion) and circulating testosterone levels and larger adipocytes than controls (Table 1).

### Phenotypic presentation

In cohort 1, 49 of the 64 women with PCOS met all three criteria for PCOS: hyperandrogenism, irregular cycles, and PCO morphology; 15 had hyperandrogenemia and PCO morphology. In cohort 2, 13 of the 21 women with PCOS met all three PCOS criteria; two had hyperandrogenemia and PCO morphology, one had hyperandrogenemia and irregular cycles, and five had irregular cycles and PCO morphology.

### Differential expression in adipose tissue

To investigate differential gene expression in adipose tissue, we used Illumina HumanHT-12 v4 Expression BeadChips to analyze subcutaneous adipose tissue from the 64 women with PCOS and the 30 controls in cohort 1. Each chip has 47,000 probes that represent well-characterized genes and unknown splice variants. A false-discovery rate (FDR) < 5% (*Q* < 0.05) was used to correct for multiple testing. After correction, 1899 probe sets, representing 1769 annotated transcripts and 1720 unique genes (*Q* < 0.05), were differentially expressed in adipose tissue from women with PCOS and controls (Table S1). Of the 1720 unique genes, 912 were down-regulated (by up to 57%) and 808 were up-regulated (by up to 58%) in women with PCOS (Table S1).

**Figure 1:**
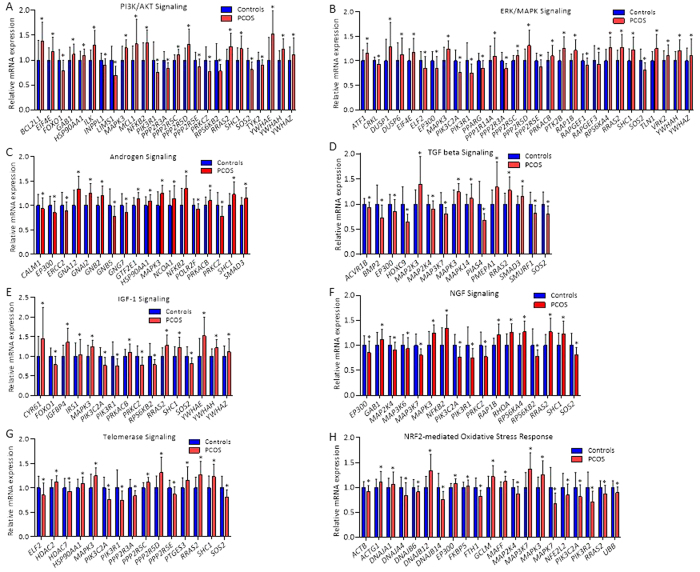
Gene sets contributing to selected significant top canonical pathways, identified by Ingenuity Pathway Analysis, of possible relevance to PCOS and T2D. Up-regulated canonical pathways: (**A**) PI3K/AKT Signaling pathway, (**B**) ERK/MAPK signaling pathway, (**C**) Androgen receptor signaling pathway, (**D**) TGF-β signaling pathway, (**E**) IGF1-1 signaling pathway, (**F**) NGF signaling pathway, and (**G**) Telomerase signaling pathway. Down-regulated canonical pathway: (**H**) NRF2-mediated oxidative stress response signaling pathway. Values are mean ± SD. *P < 0.05.

Next, all differentially expressed genes (*P* < 0.05) were analyzed with Ingenuity Pathway Analysis software to identify biological canonical pathways. All significantly up- and down-regulated gene sets are presented in Table 2. A selection of significantly up- and down-regulated canonical pathways relevant to PCOS and T2D—PI3K/AKT, ERK/MAPK-, androgen, TGF-β, IGF-1, NGF, telomerase, and NRF2-mediated oxidative stress response signaling pathways—are shown in Fig. 1A–H. All genes contributing to significantly up-regulated and down-regulated canonical pathways are presented in Tables S2 and S3, respectively.

Fifty genes whose expression in adipose tissue differed the most between women with PCOS and controls (*Q* < 0.05) are presented in Table 3. Among the most up-regulated genes (expression range 34.8% to 57.8%) we found genes associated with hormone/aromatase activity and metabolic disturbances (e.g., *CD74*, *APOLD1, CYP1B1*, and *UCP2*). Among the most down-regulated genes (expression range –28.7% to –57.4%) were those involved in metabolic disturbance, including T2D, glycerol and lipid processes (e.g.*, SLC7A10, UGP2* and *GPT*).

Next we investigated whether genes linked to PCOS, T2D, and obesity in published GWAS (*P* < 5 × 10^5^; http://www.ebi.ac.uk/gwas, accessed on August 20, 2015) were differently expressed in adipose tissue from women with PCOS versus controls (*Q* < 0.05). We found one out of nineteen PCOS candidate genes, *RAB5B*^26^, expressed at a lower level in adipose tissue from women with PCOS (Table 4). Additionally, 18 out of 597 T2D candidate genes (e.g., *PPARG*^27^, *SVEP1*^28^, and *IRS1*^29^) and five out of 1238 obesity candidate genes (e.g. *RTN4*^30^ and *LEPR*^31^) were differentially expressed in adipose tissue from women with PCOS (Table 4).

### Replication of differential mRNA expression

**Figure 2:**
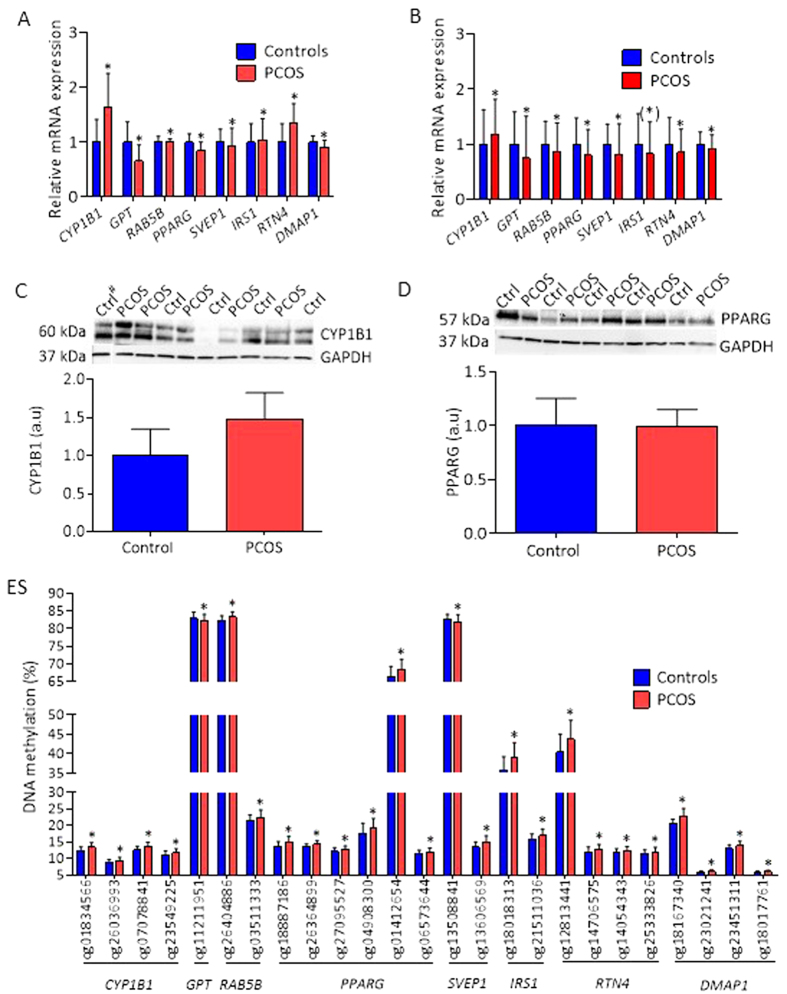
Selected genes relevant to PCOS that were differentially expressed in cohort 1 and replicated in cohort 2. (**A**) Expression data of selected genes from the Illumina HumanHT- 12 v4 Expression BeadChip array (cohort 1). (**B**) Replication of gene expression data from the array by qPCR in adipose tissue from 21 unrelated women with PCOS and 21 controls (cohort 2). Protein expression of CYP1B1 (**C**) and PPARG (**D**) in control (n=5) and PCOS (n=5) are presented. (**E**) Differential DNA methylation of biologically validated genes. Values for gene expression are mean ± SD and for proteins mean ± SEM. *P < 0.05 (*) *P* = 0.056. # Sample run on a different membrane.

We proceeded to replicate differential gene expression in cohort 2. Selected differentially expressed genes were (1) among those with largest fold change e.g.: *CYP1B1*^32^, which was up-regulated and is involved in metabolism of sex steroids and adipogenesis, and *GPT *33, which was down-regulated and is involved in glucose and amino acid metabolism; (2) among candidate genes identified in GWAS studies of PCOS^26^ (*RAB5B*, involved in regulation of intracellular vesicle transport), T2D^27^,^28^,^29^ (*PPARG, SVEP1*, and *IRS1,* involved in fatty acid storage and glucose metabolism), and obesity^30^ (*RTN4*, a multifunctional gene linked to extreme obesity); and (3) among genes with overlapping with methylation (*DMAP1*), which is involved in DNA methylation and regulation of obesity-related inflammation and cancer^34^, all with a *Q* < 0.05 (Fig. 2A, Table S4).

Six of the 20 selected genes were successfully replicated in cohort 2, as shown by significant expression differences in the same direction as in cohort 1 (Fig. 2B, Table S4). The mRNA expression of *CYP1B1* was higher and the mRNA expression of *GPT, RAB5B, PPARG*, *SVEP1*, and *DMAP1* was significantly lower in adipose tissue of women with PCOS. *IRS1* and *RTN4* were differentially expressed but in directions opposite to those in cohort 1. The expression of *CD74*, the gene with the largest increase in expression in cohort 1, was upregulated in cohort 2, although not significant (*P* = 0.061), and the expression of *ADIPOR2* was down regulated (*P* = 0.069) (Table S4).

Next we preformed western blot (WB) analyses as protein expression is more relevant for biological functions. Protein expression of CYP1B1 was higher in adipose tissue of women with PCOS compared with controls (Fig. 2C) reflecting differential gene expression obtained from BeadChip gene expression array and in the replication cohort 2 (Fig. 2A,B). Protein expression of PPARG displayed a week trend to be lower (Fig. 2D). The WB analyses are hampered by small sample size and big variations.

### Genome-wide DNA methylation analysis in adipose tissue from women with PCOS

**Figure 3:**
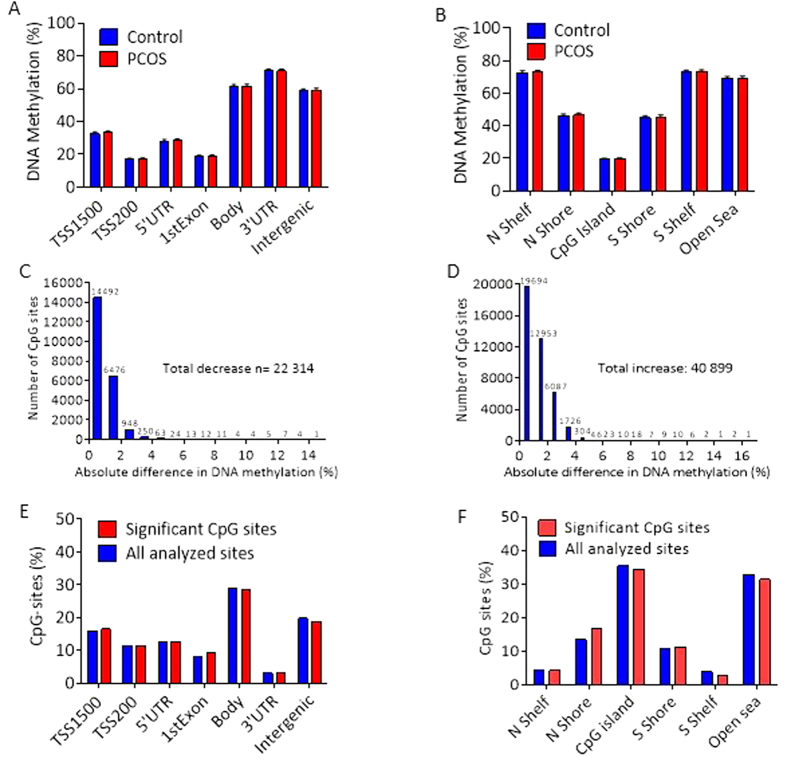
Effect of PCOS on global DNA methylation in human adipose tissue. Global DNA methylation was calculated as the average DNA methylation of all CpG sites in each annotated region on the Infinium Human Methylation 450 BeadChip presented for (**A**) the nearest gene region and (**B**) nearest CpG island region (mean ± SD). Absolute difference in DNA methylation of 63,213 individual sites divided into (**C**) sites with less methylation and (**D**) sites with more methylation in 64 women with PCOS than in 30 controls (cohort 1). Distribution of significant sites compared with all analyzed sites in relation to nearest gene region (**E**) and nearest CpG island region (**F**). TSS, proximal promotor defined as 200 or 1500 bp upstream of the transcription site; Shore, flanking region of CpG island (0–2000 bp); Shelf, regions flanking island shores (2000–4000 bp from the CpG island).

Next we used Illumina Infinium HumanMethylation450k array BeadChips to evaluate the global DNA methylation pattern in adipose tissue from women in cohort 1. After quality control and filtering, methylation data was obtained in 483,317 CpG sites. The average levels of DNA methylation for these sites were grouped based on their location in relation to the nearest gene (Fig. 3A) or in relation to CpG islands (Fig. 3B). We found no differences in average adipose tissue DNA methylation between women with PCOS and controls.

Of the 483,317 CpG sites, 63,213 (13%) were differentially methylated in adipose tissue from women with PCOS and controls (*P* < 0.05) (Table S5). This is 2.6 times more than the expected number with a *P* < 0.05 (a *P* value threshold of 5% may yield a false positive rate of 5% in the dataset) and significantly more than would be expected by chance (chi-square test *P* < 0.0001). After FDR correction, 440 CpG sites were differentially methylated (*Q* < 0.15), Table S6. The most significant CpG site was cg13496119, annotated to *LOC100129345* (*Q* = 0.046, *P* < 0.000). This uncharacterized locus of unknown function belongs to long noncoding RNAs associated with chromatin remodeling and transcriptional regulation. As judged from absolute difference in DNA methylation between cases and controls, methylation decreased at 22,314 sites (Fig. 3C) and increased at 40,899 sites (Fig. 3D). The majority of differentially methylated sites were in the gene body, intergenic regions, CpG islands, and “open sea” regions (Fig. 3E,F).

To assess the biological relevance of differently methylated genes in adipose tissue from women with PCOS and controls, we used Ingenuity Pathway Analysis software to analyze the 48,426 transcripts annotated to the 63,213 differentially methylated CpG sites (*P* < 0.05). All significantly up- and down-regulated gene sets with differential methylation are presented in (Table S7). Significantly up-regulated canonical pathways relevant to PCOS and T2D are the Wnt/β-catenin and planar cell polarity signaling pathways, which regulate glucose transporters, mitochondrial biogenesis, and adipogenesis^35^; and oxidative stress and reactive oxygen species in the macrophage signaling pathway^36^, all involved in glucose regulation, energy metabolism, and obesity. A significantly down-regulated pathway of importance is the NRF-2-mediated oxidative stress response pathway, which helps regulate a wide range of genes in response to environmental stress^37^. Importantly, the NRF-2-mediated oxidative stress response pathway was among the significant gene sets for both differential gene expression and methylation. Differentially methylated genes contributing to up-regulated and down-regulated top canonical pathways are presented in Tables S8 and S9, respectively.

Next we investigated whether genes previously linked to PCOS, T2D, and obesity in published GWAS (*P* < 5 × 10^5^; http://www.ebi.ac.uk/gwas, accessed on August 20, 2015) were differently methylated in adipose tissue from women with PCOS and controls (*Q* < 0.15). This analysis identified four out of 597 T2D candidate genes (*ARF5*^38^, *GALNTL4*^39^, *R3HDML*^40^, and *PSMD6*^40^) and one obesity candidate gene out of 1238 (*PROX1*^41^) (Table 5), but no PCOS candidate gene. DNA methylation (*P* < 0.05) in the replicated genes (Fig. 2A,B) is shown in Fig. 2E.

Finally, based on data by Chen *et al.*^42^, potentially cross-reactive probes among our differentially methylated CpG sites with *P* < 0.05 are presented in Table S10.

### Overlap and associations between gene expression and DNA methylation

Since mRNA expression can be regulated by epigenetic modifications, we next investigated whether genes whose mRNA expression in adipose tissue differed in women with PCOS and controls also differed in DNA methylation. For this analysis, we merged the mRNA expression data and the DNA methylation data (cohort 1). All genes that were differentially expressed (*P* < 0.05) were linked to corresponding methylated probes. In total, in 30 individual genes with differential mRNA expression (*Q* < 0.05), we found a corresponding change in 33 DNA methylation sites (Table S11). One of these genes, *DMAP1,* was replicated in cohort 2 and is involved in DNA methylation and regulation of obesity-related inflammation and developing cancer^34^,^42^; its mRNA expression was also replicated in cohort 2. Of note, a large number of DNA methyltransferases (*DNMTs*) genes, a family of enzymes catalyzing the transfer of a methyl group to DNA, were differentially methylated in women with PCOS compared with controls (Table S12). Next, to investigate direct correlations between gene expression and DNA methylation, we performed Spearman’s with correction for multiple testing. We included mRNA expression of the 5453 annotated transcripts differentially expressed in adipose tissue from women with PCOS (*P* < 0.05), and DNA methylation of CpG probes located within the *cis* distance 500 kb upstream and 100 kb downstream of the genes. We identified 1913 pairs of “gene-CpG probes” that were significantly correlated after correction for multiple testing (*Q* < 0.05) that corresponded to 349 unique genes (Table S13). 1057 of these correlations were positive (*Rho* = 0.37–0.61) and 856 were negative (*Rho* = −0.37–0.69).

**Figure 4:**
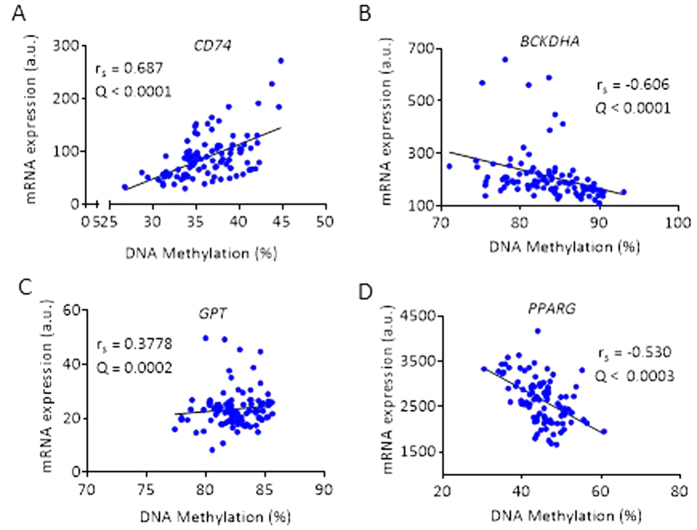
Representative illustrations of correlations of gene expression with DNA methylation in adipose tissue from cohort 1. Expression *CD74* correlated positively with DNA methylation (**A**); *BCKDHA* correlated negatively with DNA methylation (**B**); *GPT* correlated positively with DNA methylation (**C**); and *PPARG* correlated negatively with DNA methylation (**D**). Spearman rank correlation with FDR corrections.

Several probes of *CD74*, the gene with the highest increase in expression difference, showed a strong positive correlation with DNA methylation (*Rho* = 0.611–0.687; *Q* < 0.05) (Table S13). The CD74 molecule is associated with class II major histocompatibility complex (MHC) class II invariant chain that is involved in regulation of adipogenesis and inflammation^43^. The gene with the strongest negative correlation with DNA methylation was the branched chain keto acid dehydrogenase E1 alpha polypeptide provided (*BCKDHA*) gene (Table S13). This gene is involved in metabolic signaling and insulin resistance^44^. Representative correlation between mRNA expression and DNA methylation of *CD74, BCKDHA, GPT*, and *PPARG* are shown in Fig. 4A–D.

### Associations between hyperandrogenemia, glucose homeostasis, or adipocyte size and gene expression or DNA methylation in adipose tissue

**Figure 5:**
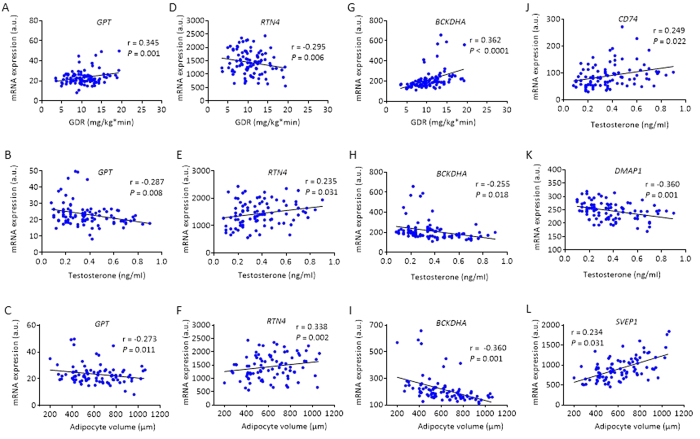
Correlations of GDR, adipocyte size, and circulating testosterone level with gene expression in adipose tissue from cohort 1. Expression of *GPT* correlated positively with GDR (**A**), negatively with circulating testosterone (**B**), and with adipocyte volume (**C**); *RTN4* correlated negatively with GDR (**D**), positively with testosterone (**E**), and with adipocyte volume (**F**); *BCKDHA* correlated positively with GDR (**G**), negatively with circulating testosterone (**H**), and with adipocyte volume (**I**); *CD74* correlated positively with circulating testosterone (**J**); *DMPA1* correlated negatively with circulating testosterone (**K**); and *SVEP1* correlated positively with adipocyte volume (**L**). Pearson’s partial correlation.

Hyperandrogenemia and insulin resistance are the strongest clinical features of PCOS, and adipocytes size is a strong marker for insulin resistance in women with PCOS^9^. Therefore, we tested whether the GDR measured by euglycemic hyperinsulinemic clamp, circulating testosterone measured by LC-MS/MS, or adipocyte size was associated with (1) genes with largest fold changes *CD74, CYP1B1* and *GPT*; (2) candidate genes identified in GWAS studies of PCOS (*RAB5B*), T2D (*PPARG, SVEP1* and *BCKDHA*), and obesity (*RTN4*); and, (3) a gene overlapping with methylation (*DMAP1*). With Spearman’s we found a significantly positive correlation between GDR and the expression of *RAB5B* (r_s_ = 0.211), *PPARG* (r_s_ = 0.426), and *BCKDHA* (r_s_ = 0.561), and negative correlation with *CD74* (r_s_ = −0.281), *SVEP1* (r_s_ = −0.421), and *CYP1B1* (r_s_ = −0.325). Circulating testosterone correlated positively with the expression of *CD74* (r_s_ = 0.356)*, CYP1B1* (r_s_ = 0.219) and *RTN4* (r_s_ = 0.261), and negatively with the expression of *GPT* (r_s_ = −0.317)*, PPARG* (r_s_ = −0.269)*, BCKDHA* (r_s_ = −0.463), and *DMAP1* (r_s_ = −0.339). Adipocyte size was positively correlated with *CD74* (r_s_ = 0.425)*, SVEP1* (r_s_ = 0.525), *RTN4* (r_s_ = 0.217), and *CYP1B1* (r_s_ = 0.284), and negatively associated with *PPARG* (r_s_ = −0.449) and *BCKDHA* (r_s_ = −0.587). Correlations that remained significant after adjustments for BMI are presented in Fig. 5A–L.

## Discussion

This study shows that genome-wide mRNA expression in subcutaneous adipose tissue is altered in women with PCOS and that several genes are associated with insulin resistance, adipocyte size, and hyperandrogenemia. Among the top up- and down-regulated canonical pathways relevant to PCOS and T2D, we found gene sets representing the PI3K/AKT, ERK/MAPK, androgen, TGF-β, telomerase, and NRF2-mediated oxidative stress response signaling pathways. We also demonstrate for the first time that genome-wide DNA methylation alterations in adipose tissue were larger than expected. However, correction for multiple testing reduced the number of differentially methylated sites to 440. Also, we found more than 1900 pairs of “gene-CpG probes” that were significantly correlated after correction for multiple testing and corresponded to 349 unique genes. Since PCOS is a polygenic disease, the combined effect of several modest changes in DNA methylation might contribute to the pathogenesis of PCOS. This speculation is supported by other studies demonstrating a relative modest difference (0.13% to 11%) in DNA methylation in different target tissues^45^. Also, an absolute change of a few percent could make a relatively large difference, as we found in a recent study in which the fold change in DNA methylation ranged from 0.54 to 1.84 between the groups^45^. Consistent with these observations, differences of DNA methylation in the present study ranged from 0.24 to 6.28.

We identified several genes with differential mRNA expression and corresponding changes in DNA methylation, indicating that altered DNA methylation may have the potential to influence the expression of corresponding genes. Genes with the largest expression differences e.g. *CD74, CYP1B1,* and *GPT* were strongly associated with DNA methylation after FDR corrections. These genes are involved in adipogenesis and inflammatory response (*CD74*)^46^, in metabolism of sex steroids and adipogenesis (*CYP1B1)*^47^, and in glucose and amino acid metabolism (*GPT*)^33^. In candidate genes for PCOS (*RAB5B*), T2D (*PPARG* and *SVEP1*), and methylation (*DMAP1*) we found that a decrease in mRNA expression was paralleled with an increase in DNA methylation, consistent with previous functional *in vitro* studies demonstrating that an increase in DNA methylation reduces transcriptional activity^48^,^49^. Many of the up-regulated genes had a parallel increase in DNA methylation. One possible explanation is that these CpG sites are in the gene body region, as evidenced by the over-representation of differentially methylated sites we found in this region. Importantly, DNA methylation in the gene body positively affects gene expression^50^.

Although changes in mRNA expression were more pronounced than changes in DNA methylation, we found a large number of differentially expressed genes and methylated CpG sites that were significantly correlated after FDR correction. Of note, changes in methylation can be targets for other kind of transcriptional regulation, microRNAs, or histone modifications. There may also be an interaction between nongenetic, epigenetic, and genetic factors affecting gene expression and subsequent development of polygenic diseases such as PCOS, T2D, and obesity^51^,^52^,^53^,^54^. Support for a genetic effect comes from a study demonstrating that T2D-related single nucleotide polymorphisms (SNPs) are CpG-SNPs, which affect DNA methylation and gene expression in human pancreatic cells^55^, and from studies of multiple tissues from twins^56^,^57^. Of note, studies in twins indicate that genetic influences explain more than 70% of PCOS pathogenesis^58^. Nevertheless, recent GWAS have not identified SNPs that could explain this estimated heritability^26^,^59^, which therefore might reflect contributions of epigenetic variations.

The first GWAS on Han Chinese populations identified more than 10 susceptibility loci for PCOS, including *RAB5B*^26^,^59^. This gene encodes the Ras-related protein RAB5B. Although the functional role of this PCOS susceptible gene in adipose tissue is unknown, *RAB5B* expression was down-regulated both in the discovery cohort and in the replication cohort and was accompanied by an up-regulation in DNA methylation. Genes acting through Rab5b-dependent post-translational regulation of β1/β2 integrins^60^, indicates a functional role of *RAB5B* in a target tissue.

A very recent GWAS of women of European ancestry who had a classic PCOS phenotype (hyperandrogenism and irregular menstruation) identified two novel genetic susceptibility loci that mapped to chr 8p32.1 and chr 11p14.1^61^. Interestingly, we found differential DNA methylation in all four genes that mapped to the chr 8p32.1 PCOS candidate locus (*NEIL2, FDFT1, GATA4*, and *CTSB*) and differential expression of *FDF1* and *CTSB*. Further, we found differential gene expression and DNA methylation of one of the most promising functional candidate genes—*ARL14EP*, which maps to chr 11p14.1—but no changes in the *FSHB* expression or methylation in adipose tissue from women with PCOS. In the GWAS study mentioned above, there was no evidence of transcriptional activation and microarray analysis revealed low levels in adipocytes.

We also found that two T2D susceptibility genes, *PPARG*^27^ and *SVEP1*^28^, followed the same pattern of decreased expression and increased DNA methylation, and both showed a strong negative correlation between “gene-CpG probes” after multiple corrections. *PPARG*, a master regulator of adipocyte differentiation, directly controls the expression of genes involved in lipid transport and metabolism, adipokine production, and insulin signaling^62^; it is a target for insulin-sensitizing drugs such as glitazones, which improve plasma glucose maintenance in patients with T2D^63^. Previously we found higher levels of DNA methylation of *PPARG* in adipose tissue in subjects with T2D than in controls^21^. *PPARG* also protects against vascular calcification by inducing the expression of secreted frizzled-related protein-2, a Wnt5a antagonist. Targeting the Wnt/β-catenin signaling pathway may have clinical implications in the context of common complications of atherosclerosis, T2D and obesity^64^,^65^. Of interest, we found DNA methylation activated gene sets in the Wnt/β-catenin signaling pathway in adipose tissue in the present study. Previous studies have implicated that Wnt signaling activation of β-catenin, a negative co-activator, and *PPARG* might dysregulate adipogenesis^66^. *PPARG* gene expression was positively associated with insulin sensitivity (GDR) and negatively associated with adipocyte size and testosterone, although these associations did not survive after adjustment for BMI. Further, many of the significant canonical pathways included gene sets with differentially methylated genes *or* with differential gene expression in human adipose tissue that are similar to pathways detected in adipose tissue of prenatal androgenized rhesus monkeys^67^ (e.g., the Wnt/β-catenin and TGF-β signaling pathways). These findings support the hypothesis that maternal androgen excess causes an unfavorable intrauterine environment and leads to epigenetic changes that contribute to the development of insulin resistance and altered molecular signaling pathways in adipose tissue of women with PCOS.

*SVEP1*, another gene associated with T2D^28^, encodes a cell-adhesion molecule expressed by cells related to skeletal tissues. Hypermethylation of *SVEP1* decreases its expression^68^, consistent with our finding of decreased mRNA expression and increased DNA methylation of *SVEP1* in adipose tissue in women with PCOS; however, *SVEP1* mRNA expression correlated negatively with adipocyte volume after adjustment for BMI.

*IRS1*, a substrate of insulin receptor tyrosine kinase, have a central role in the insulin stimulated signaling and is a candidate gene of T2D^69^. We were not able to replicate the expression found in cohort 1. Instead there was a trend towards decreased mRNA expression of *IRS1* was (*P* = 0.058) in cohort 2. Of note, cohort 1 includes normal, overweight and obese subjects and all these analyses were corrected for BMI. The decrease in *IRS1* mRNA expression may reflect that the replication cohort was overweight – obese. This is further supported by the fact that when dividing cohort 1 into lean *vs* overweight – obese, the *IRS1* mRNA expression was lower among the overweight – obese women (mean ± SD) 318.2 ± 113.4 *vs* 232.4 ± 71.8, *P* < 0.001). This phenomenon has previously been demonstrated in e.g. Pima Indians there *IRS1* mRNA expression is approximately 1.8 fold lower in adipocytes from obese subjects compared with normal weight subjects^70^. The results in the present study is in agreement with a recent study demonstrating that the insulin signaling pathway in subcutaneous adipose tissues is not the major contributor to the pathogenesis of PCOS^71^.

DNA methylation is important in many biological processes, such as regulation of transcription, genomic imprinting, chromatin structure, and silencing of repetitive DNA elements^72^. Abnormal DNA methylation is common in human cancers and contributes to tumorigenesis^73^. DNA methylation in mammalian cells is carried out by DNMTs. DNMT1-associated protein (DMAP1) has an intrinsic repressive activity and helps maintain DNA methylation in a heritable manner^74^. Interestingly, a large number of *DNMT*s were differentially methylated in adipose tissue form women with PCOS. These finding further support the hypothesis that maternal androgen excess^75^ can induce epigenetic changes^57^.

Adipose tissue is composed of many cell types, and changes in cell composition may have contributed to the differences in gene expression and DNA methylation we observed. Previously, we showed that there is no major impact of cellular composition or inflammatory response on observed associations in DNA methylation in adipose tissue and BMI and HbA1c^53^. Although adipocyte isolation *per se* may affect gene expression and DNA methylation, it would be of interest to investigate genome-wide gene expression and DNA methylation in adipocytes from different fat depots. Future studies should also investigate the role of additional epigenetic mechanisms, including miRNAs and histone modifications which may be of importance in PCOS. Analyzes in the present study is limited to subcutaneous adipose tissue. Visceral fat accumulation is an independent risk factor of T2D^76^. The visceral fat depot is drained by the portal venous system leading to a direct supply of free fatty acids, and may therefore be considered as more metabolic active^77^. However, different genes may have different functions in the two depots; e.g. *PPARG* have a twofold higher mRNA expression in subcutaneous than in visceral adipose tissue in lean/overweight subjects^78^. In contrast, in obese subjects, mRNA expression of *PPARG* was similar in the two depots. Of note, in the present study including both lean and overweight/obese women with PCOS displayed lower *PPARG* expression independent of BMI. Women with PCOS display a “male like” fat distribution with abdominal (subcutaneous and visceral) fat accumulation^79^. Few studies have investigated differences between gene expression in subcutaneous and visceral fat depots in women with PCOS with no major differences^15^,^80^. Another limitation is the cross sectional design and that we have only 30 controls compared with 64 cases, although to date this study is the largest investigating genome wide DNA methylation and expression in women with PCOS.

In conclusion, we identified a large number of genes and pathways that are affected in adipose tissue from women with PCOS. We also identified specific DNA methylation pathways that may affect mRNA expression. Together, these novel findings show that women with PCOS have multiple transcriptional and epigenetic changes in adipose tissue that are relevant for development of the disease.

## Material and Methods

### Study participants

Cohort 1 consisted of 64 women with PCOS and 30 controls who had successful subcutaneous adipose tissue biopsies and were previously described in detail^9^. Case-control cohort 2 consisted of 21 women with PCOS and 21 controls matched pairwise for age, weight, and BMI were included for replication of the findings in cohort 1.

All women provided oral and written informed consent. The two studies were conducted at the Sahlgrenska Academy, University of Gothenburg and at Sahlgrenska University Hospital, Gothenburg, Sweden, in accordance with the Declaration of Helsinki and were approved by the Regional Ethical Review Board of the University of Gothenburg. After all relevant clinical information was obtained, samples were coded and anonymized.

Subject recruitment, exclusion and inclusion criteria are described in detail in Supplementary Material and Methods.

### Clinical examination

Body weight and height were measured in subjects wearing light clothing; body mass index was calculated as kg/m^2^. Waist circumference was measured between the lower rib and iliac crest. In controls, blood samples were obtained in the morning after an overnight fast during the early follicular phase (days 1–7 of the menstrual cycle) to match the hormonal milieu of PCOS subjects and to avoid the preovulatory estrogen rise. Fasting blood samples were collected independently of cycle day in women with PCOS as the majority had oligo/anovulation. Subcutaneous abdominal adipose tissue biopsies were obtained under local anesthesia and immediately isolated for measurement of adipocyte size^9^ or snap frozen in liquid nitrogen and stored at −80 °C. Whole-body glucose homeostasis was measured by euglycemic hyperinsulinemic clamp with calculation of GDR, and by calculation of HOMA-IR and HOMA-B^9^.

### Biochemical analyses

Plasma glucose was measured at 37 °C with an enzymatic photometric method (Roche Diagnostics, Mannheim, Germany) in cohort 1 and by One Touch Ultra2 (LifeScan) in cohort 2. Serum insulin was measured with an immunometric two-step sandwich method and chemiluminescence (Advia Centaur Insulin ReadyPack; Bayer HealthCare). Estrogen and testosterone were measured by gas chromatography-tandem mass spectrometry, and SHBG was analyzed by chemiluminescent microparticle immunoassay as described^10^,^81^.

### RNA and DNA Extraction from Adipose Tissue

For gene expression array studies, RNA was extracted with the RNeasy Lipid tissue Mini Kit (Qiagen). For methylation array studies, DNA was isolated with the QIAamp DNA Mini Kit (Qiagen). Nucleic acid concentrations and purity were estimated with a NanoDrop spectrophotometer (Thermo Scientific). DNA integrity was also checked by gel electrophoresis, and RNA quality was determined with an automated electrophoresis station (Experion, Bio-Rad).

### Expression Arrays and quantitative real-time PCR

To assess the global mRNA expression profile in adipose tissue, we analyzed isolated mRNA with high abundance with microarray HumanHT-12 v4 Expression BeadChip (Illumina). cRNA synthesis, including biotin labeling, was carried out using Illumina TotalPrep RNA Amplification Kit (Life Technologies & Invitrogen) according to manufacturer’s recommendations. Biotin-cRNA complex was then fragmented and hybridized to the probes on the Illumina BeadChip array. Probes were hybridized and stained with streptavidin-Cy3 before visualization with Illuminas HiScan fluorescence camera. The Oligo package from Bioconductor was used to compute Robust Multichip Average expression measures^82^.

In cohort 2, quantitative real-time PCR analysis of selected genes (Table S4) was validated with custom TaqMan gene expression array micro fluid cards (Life Technologies) i.e. the replication cohort. Samples were run in duplicate; the amount of cDNA in each loading port was equivalent to 100 ng of mRNA. The arrays were run according to the manufacturer’s protocol with a QuantStudio 7 Flex Real-time PCR System and QuantStudio 7 software (Life Technologies). Candidate reference genes *(GAPDH, LRP10*, and *CLN3*) were validated with NormFinder algorithm; from this algorithm, the combination of *LRP10* and *CLN3* was used as the reference control. Gene expression values were calculated with the ΔΔC_q_ method (i.e., RQ = 2^−ΔΔCq^).

### Immunoblot Analysis

To analyze protein expression in adipose tissue homogenates we used antibodies against CYP1B1 (AV51761, Sigma-Aldrich, Stockholm Sweden) and PPARG (ab191407, Abcam, Cambride, UK). Briefly, samples were centrifuged at 13,000 g for 10 min at 4 °C and protein concentration was determined with a Direct Detect™ spectrometer (Millipore, Billerica, USA). 50 μg of total protein was loaded on Criterion™ TGX (Tris-Glycine eXtended) Stain-Free™ precast gels (Bio-Rad) and transferred to nitrocellulose midi-membrane in the Trans-Blot^®^ Turbo™ Transfer System (Bio-Rad). GAPDH was used as a loading control and for normalization.

### DNA Methylation Arrays

Genome-wide DNA methylation in adipose tissue was analyzed with Illumina Infinium HumanMethylation450k array BeadChips. The array contains 485,577 cytosine probes covering 21,231 (99%) RefSeq genes^83^. A Zymo Methylation Kit (D5001-D5002, Zymo Research) was used to convert genomic DNA to the bisulfite-modified DNA. Briefly, gDNA (500 ng) of high quality was fragmented and hybridized on the BeadChip, and the intensities of the signal were measured with a HiScanQ scanner (Illumina). The methylation values for each CpG site are presented as a β-value ranging from 0 (unmethylated) to 100% (completely methylated).

The bioinformatics analyses were performed as described^21^,^53^. In brief, Y chromosome probes, rs-probe, and probes with average detection *p*-value > 0.01 were removed. After quality control and filtering, methylation data was obtained in 483,317 CpG sites. Beta-values were converted to M-values (M = log2 (β/(1 − β), which were used for all data analyses. Data were then quantile normalized and batch corrected with COMBAT^84^. A linear regression model was used to identify differences in DNA methylation between women with PCOS and controls. To improve interpretation, after all the preprocessing steps, data were reconverted to beta-values, which are presented in tables and figures.

### Ingenuity pathway analysis

Ingenuity Pathway Analysis (Qiagen) was used for core analyses of data from the gene expression array and DNA methylation array so we could interpret the outcome and make predictions based on known libraries. Fold change and *Q*- and *P*-values were calculated before data entry, and thereafter uploaded for Ingenuity Pathway Analysis. *Q* < 0.05 was used as the cut-off for gene expression and *P* < 0.05 for DNA methylation. Top canonical pathways are based on the *P*-value overlap of genes in our dataset with molecules in the canonical pathway.

### Statistics

Differences between clinical characteristics in cohort 1 were based on ANCOVA with adjustment for age and BMI. Fisher’s permutation test was used for comparisons of clinical characteristics in cohort 2; no adjustments for age and BMI were needed, as the cases and controls were matched for age, weight, and BMI. DNA methylation differences between cases and controls in cohort 1 were based on linear regression including batch, age and BMI. The Mann-Whitney U test was used for expression analyses. FDR was used to correct for multiple testing in the analyses of gene and methylation arrays. The chi-square test was used to calculate whether the differentially methylated sites were more than the expected number by chance. Also, differentially expressed genes with a *Q* < 0.05 were merged with corresponding probes in the methylation data. For these analyses, a CpG site was annotated to the nearest gene. Correlations in women with PCOS plus controls (cohort 1) between DNA methylation and gene expression of selected genes and GDR and testosterone were analyzed by Spearman´s correlation with correction for multiple testing. Data are presented as mean ± SD.

## Additional Information

**How to cite this article**: Kokosar, M. *et al.* Epigenetic and Transcriptional Alterations in Human Adipose Tissue of Polycystic Ovary Syndrome. *Sci. Rep.* **6**, 22883; doi: 10.1038/srep22883 (2016).

## Supplementary Material

Supplementary Information

Supplementary Table S1

Supplementary Table S2

Supplementary Table S3

Supplementary Table S4

Supplementary Table S5

Supplementary Table S6

Supplementary Table S7

Supplementary Table S8

Supplementary Table S9

Supplementary Table S10

Supplementary Table S11

Supplementary Table S12

Supplementary Table S13

## Figures and Tables

**Table 1:** Clinical characteristics of women with polycystic ovary syndrome and controls in cohorts 1 and 2.

Cohort 1	Controls (n = 30)	PCOS (n = 64)	*P* value^a^
Age (years)	27.6 ± 3.54	30.0 ± 4.4	NA
Anthropometry			
BMI (kg/m^2^)	24.8 ± 5.0	28.4 ± 7.1	NA
Waist-to-Hip-Ratio	0.79 ± 0.06	0.84 ± 0.07	0.027
PCOS characteristics, n (%)			
Oligo/amenorrhea	0	76% (55)	NA
Hirsutism	0	75% (54)	NA
Acne (yes/no)	0	64% (46)	NA
Hormones			
Estradiol (pg/ml)	38.9 ± 18.2	73.9 ± 40.3	< 0.000
Testosterone (ng/ml)	0.21 ± 0.08	0.45 ± 0.18	< 0.000
SHBG (nmol/l)	67.8 ± 29.6	39.3 ± 21.3	< 0.000
Metabolic measures			
HOMA-IR	1.22 ± 0.82	2.48 ± 2.16	0.008
HOMA-B	152.5 ± 113.2	203.3 ± 159.6	0.522
GDR (mg/kg x min)	12.58 ± 3.45	9.69 ± 3.16	0.005
Hba1c (mmol/mol)	28.73 ± 5.94	31.56 ± 3.07	0.016
Adipocyte size (μm)	93.2 ± 9.87	100.2 ± 11.45	0.153
**Cohort 2**	**Controls (n = 21)**	**PCOS (n = 21)**	***P* value**^b^
Age (years)	29.8 ± 6.4	31.2 ± 5.6	0.520
Anthropometry			
BMI (kg/m^2^)	30.4 ± 3.62	31.2 ± 4.12	0.534
Waist-to-Hip-Ratio	0.82 ± 0.05	0.85 ± 0.08	0.155
PCOS characteristics, n (%)			
Oligo/amenorrhea	0	85% (18)	NA
Hirsutism	0	67% (14)	NA
Acne (yes/no)	0	43% (9)	NA
Hormones			
Estradiol (pg/ml)	57.3 ± 41.8	87.12 ± 63.2	0.080
Testosterone (ng/ml)	0.25 ± 0.09	0.49 ± 0.30	< 0.001
SHBG (nmol/l)	39.5 ± 25.0	38.9 ± 16.2	0.819
Metabolic measures			
HOMA-IR	2.20 ± 1.11	3.18 ± 2.05	0.061
HOMA-B	121.5 ± 62.9	196.9 ± 132.7	0.020
GDR (mg/kg x min)	6.73 ± 2.84	6.17 ± 3.11	0.549
Hba1c (mmol/mol)	31.8 ± 2.91	31.6 ± 2.55	0.911
Adipocyte size (μm)	110.5 ± 8.03	116.7 ± 9.78	0.030

Values are mean ± standard deviation (SD). BMI, body mass index; SHBG, sex hormone binding globulin; LH, luteinizing hormone; FSH, follicle-stimulating hormone; HOMA, homeostatic model assessment; GDR, glucose disposal rate.

^a^ANCOVA with adjustment for age and BMI.

^b^Fisher’s permutation test.

**Table 2:** Significant gene sets with differential gene expression in adipose tissue from women with PCOS versus controls. IPA analyses with *Q* < 0.05.

Pathway	Regulated/total	Ratio	Z-score	*P* value	Signaling Pathway Category	Top Function & Diseases
**Up-regulated pathways**						
PI3K/AKT Signaling	25/113	0.221	2.132	1.72E-5	Cancer; Cellular Growth, Proliferation and Development; Intracellular and Second Messenger Signaling	Cell Death and Survival; Cancer; Cell Cycle
ILK Signaling	28/152	0.184	2.887	1.79E-4	Cellular Growth, Proliferation and Development	Cellular Movement; Cancer; Cardiovascular System Development and Function
ERK/MAPK Signaling	29/167	0.174	1.347	3.92E-4	Cancer; Intracellular and Second Messenger Signaling	Cancer; DNA Replication, Recombination, and Repair; Cell Cycle
Cholecystokinin/Gastrin-mediated Signaling	19/93	0.204	4.359	5.03E-4	Neurotransmitters and Other Nervous System Signaling	DNA Replication, Recombination, and Repair; Developmental Disorder; Cellular Development
ERK5 Signaling	14/62	0.226	3.162	9.60E-4	Intracellular and Second Messenger Signaling	DNA Replication, Recombination, and Repair; Cancer; Developmental Disorder
p70S6K Signaling	20/107	0.187	1.698	1.18E-3	Cellular Growth, Proliferation and Development; Cellular Stress and Injury	Cellular Function and Maintenance; Cell Death and Survival; Post-Translational Modification
Role of NFAT in Cardiac Hypertrophy	26/160	0.163	3.545	2.06E-3	Cardiovascular Signaling; Disease-Specific Pathways	Cardiac Hypertrophy; Cardiovascular Disease; Developmental Disorder
Cardiac Hypertrophy Signaling	29/191	0.152	4.707	3.35E-3	Cardiovascular Signaling; Disease-Specific Pathways	Cardiac Hypertrophy; Cardiovascular Disease; Developmental Disorder
Androgen Signaling	18/103	0.175	2.333	4.35E-3	Nuclear Receptor Signaling	ene Expression; Cell Death and Survival; Cellular Development
TGF-β-Signaling	15/80	0.188	1.508	4.51E-3	Cellular Growth, Proliferation and Development; Growth Factor Signaling; Ingenuity Toxicity List Pathways	Gene Expression; Embryonic Development; Organismal Development
IGF-1 Signaling	16/92	0.174	2.53	7.27E-3	Cellular Growth, Proliferation and Development; Growth Factor Signaling	Cancer; Cellular Development; Hematological System Development and Function
VEGF Signaling	15/85	0.176	3.606	8.03E-3	Cellular Growth, Proliferation and Development; Growth Factor Signaling	Cardiovascular System Development and Function; Cell Cycle; Cell Death and Survival
Phospholipase C Signaling	29/207	0.14	4.69	1.04E-2	Intracellular and Second Messenger Signaling	Cell Signaling; Molecular Transport; Vitamin and Mineral Metabolism
Acute Myeloid Leukemia Signaling	13/72	0.181	3.051	1.08E-2	Cancer; Disease-Specific Pathways	Cellular Development; Cellular Growth and Proliferation; Hematological System Development and Function
Cardiac β-adrenergic Signaling	18/113	0.159	0.302	1.14E-2	Cardiovascular Signaling	Skeletal and Muscular System Development and Function; DNA Replication, Recombination, and Repair; Molecular Transport
NGF Signaling	17/105	0.162	4.123	1.18E-2	Growth Factor Signaling; Neurotransmitters and Other Nervous System Signaling	Cancer; Cellular Growth and Proliferation; Cellular Development
Gαq Signaling	20/131	0.153	4.243	1.26E-2	Intracellular and Second Messenger Signaling	Post-Translational Modification; Lipid Metabolism; Small Molecule Biochemistry
Integrin Signaling	25/175	0.143	4.379	1.31E-2	Cell Cycle Regulation; Cellular Growth, Proliferation and Development; Intracellular and Second Messenger Signaling	Cell Morphology; Cellular Assembly and Organization; Cellular Function and Maintenance
AMPK Signaling	20/133	0.15	0.832	1.47E-2	Cellular Growth, Proliferation and Development; Intracellular and Second Messenger Signaling	Energy Production; Lipid Metabolism; Small Molecule Biochemistry
Telomerase Signaling	15/91	0.165	1.155	1.48E-2	Apoptosis; Cancer	Cellular Assembly and Organization; Cellular Function and Maintenance; Cancer
Ceramide Signaling	13/75	0.173	2.496	1.51E-2	Apoptosis; Cell Cycle Regulation	Cell Death and Survival; Cell Signaling; Renal Necrosis/Cell Death
mTOR Signaling	24/170	0.141	2.183	1.70E-2	Cellular Growth, Proliferation and Development	Cell Morphology; Protein Synthesis; Cellular Function and Maintenance
fMLP Signaling in Neutrophils	16/102	0.157	3.742	1.89E-2	Cellular Immune Response; Cytokine Signaling	Cell-To-Cell Signaling and Interaction; Cellular Function and Maintenance; Inflammatory Response
CXCR4 Signaling	19/128	0.148	4	1.94E-2	Cellular Immune Response; Cytokine Signaling	Cell Morphology; Cell-To-Cell Signaling and Interaction; Cellular Function and Maintenance
Melanocyte Development and Pigmentation Signaling	13/79	0.165	3.606	2.26E-2	Cellular Growth, Proliferation and Development; Growth Factor Signaling	Cellular Development; Tissue Development; Hair and Skin Development and Function
Colorectal Cancer Metastasis Signaling	28/211	0.133	5	2.27E-2	Cancer; Disease-Specific Pathways	Cell Death and Survival; Cell Cycle; Cellular Development
Relaxin Signaling	17/115	0.148	3	2.70E-2	Growth Factor Signaling; Organismal Growth and Development	Cellular Development; Cellular Growth and Proliferation; Organ Development
Neurotrophin/TRK Signaling	11/65	0.169	2.714	2.85E-2	Growth Factor Signaling; Neurotransmitters and Other Nervous System Signaling	Cellular Growth and Proliferation; Cell Cycle; Cell Morphology
SAPK/JNK Signaling	14/90	0.156	2–496	2.87E-2	Apoptosis	Cell Signaling; Post-Translational Modification; Cell Death and Survival
Role of CHK Proteins in Cell Cycle Checkpoint Control	9/50	0.18	1.134	3.17E-2	Cell Cycle Regulation; Cellular Stress and Injury	Cell Cycle; DNA Replication, Recombination, and Repair; Cell Death and Survival
Regulation of eIF4 and p70S6K Signaling	19/135	0.141	1.155	3.20E-2	Cell Cycle; DNA Replication, Recombination, and Repair; Cell Death and Survival	Protein Synthesis; Cell Cycle; Cancer
IL-1 Signaling	18/83	0.157	3.162	3.26-2	Cytokine Signaling	Cell-mediated Immune Response; Cellular Development; Cellular Function and Maintenance
Role of NFAT in Regulation of the Immune Response	21/154	0.136	3.873	3.41E-2	Cellular Immune Response; Humoral Immune Response; Intracellular and Second Messenger Signaling	Cellular Development; Hematological System Development and Function; Hematopoiesis
Sphingosine-1-phosphate Signaling	14/93	0.151	3.606	3.68E-2	Intracellular and Second Messenger Signaling	Cellular Development; Embryonic Development; Organismal Development
VEGF Family Ligand-Receptor Interactions	11/69	0.159	3.162	4.20E-2	Cellular Growth, Proliferation and Development; Growth Factor Signaling	Cell Cycle; Organismal Development; Embryonic Development
FLT3 Signaling in Hematopoietic Progenitor Cells	11/70	0.157	3.317	4.60E-2	Cytokine Signaling	Cell Cycle; Cellular Development; Hematological System Development and Function
p38 MAPK Signaling	15/105	0.143	2.673	4.70E-2	Cellular Immune Response; Cellular Stress and Injury; Cytokine Signaling; Humoral Immune Response; Intracellular and Second Messenger Signaling	Gene Expression; Cell Death and Survival; Cellular Development
Melatonin Signaling	10/62	0.161	2.828	4.77E-2	Neurotransmitters and Other Nervous System Signaling	Cell Signaling; Molecular Transport; Small Molecule Biochemistry
GDNF Family Ligand-Receptor Interactions	10/62	0.161	3	4.77E-2	Growth Factor Signaling; Neurotransmitters and Other Nervous System Signaling	Cellular Development; Cellular Growth and Proliferation; Nervous System Development and Function
Production of Nitric Oxide and Reactive Oxygen Speaces in Macrophages	21/160	0.131	2.236	4.85E-2	Cellular Immune Response	Cell Signaling; Small Molecule Biochemistry; Free Radical Scavenging
CNTF Signaling	8/46	0.174	2.828	4.96E-2	Cellular Growth, Proliferation and Development; Cytokine Signaling; Neurotransmitters and Other Nervous System Signaling	Cellular Development; Cellular Growth and Proliferation; Hematological System Development and Function
Tec Kinase Signaling	19/142	0.134	3.742	4.98E-2	Intracellular and Second Messenger Signaling	Cell-To-Cell Signaling and Interaction; Cellular Function and Maintenance; Inflammatory Response
**Down-regulated pathways**						
HIPPO Signaling	18/77	0.234	−1,069	1.20E-4	Organismal Growth and Development	Cancer; Organismal Injury and Abnormalities; Cell Cycle
NRF2-mediated Oxidative Stress Response	23/136	0.141	−3,606	1.92E-2	Cellular Stress and Injury; Ingenuity Toxicity List Pathways	Cell Death and Survival; Post-Translational Modification; Organismal Survival
PPARα/RXRα Activation	21/152	0.138	−2,357	3.01E-2	Ingenuity Toxicity List Pathways; Nuclear Receptor Signaling	Energy Production; Lipid Metabolism; Small Molecule Biochemistry

**Table 3:** Fifty individual genes with the largest mRNA expression differences in adipose tissue between PCOS (n = 64) and controls (n = 30) (*Q* < 0.05) including uncharacterized (LOC) genes.

Up-regulated genes						
Symbol	Gene Description	Controls mean ± SD	PCOS mean ± SD	Difference (%)	*p* value	*Q*value
*CD74*	CD74 molecule, major histocompatibility complex, class II invariant chain	59.4 ± 1.4	93.7 ± 1.5	57.8	8E-06	1E-03
*SNORD48*	small nucleolar RNA, C/D box 48	11.2 ± 1.4	17.5 ± 1.5	56.4	3E-07	1E-04
*APOLD1*	apolipoprotein L domain-containing 1	217.2 ± 1.6	330.9 ± 1.6	52.3	9E-06	1E-03
*IDO1*	indoleamine 2,3-dioxygenase 1	18 ± 1.5	26.9 ± 1.6	49.6	9E-04	2E-02
*MYL9*	myosin, light chain 9, regulatory	267.2 ± 1.3	397.2 ± 1.3	48.6	2E-07	7E-05
*NRCAM*	neuronal cell-adhesion molecule	102.3 ± 1.5	151.2 ± 1.5	47.8	4E-04	1E-02
*CYP1B1*	cytochrome P450, family 1, subfamily B, polypeptide 1	61.3 ± 1.4	89.8 ± 1.4	46.3	1E-05	1E-03
*CYR61*	cysteine-rich, angiogenic inducer, 61	169.7 ± 1.5	248 ± 1.7	46.1	3E-04	1E-02
*AZIN1*	antizyme inhibitor 1	33.5 ± 1.6	48.9 ± 1.7	45.7	8E-04	2E-02
*STC1*	stanniocalcin 1	28 ± 1.5	40.4 ± 1.6	44.3	3E-03	4E-02
*NEK7*	NIMA-related kinase 7	40.8 ± 1.4	58 ± 1.4	42.2	1E-05	1E-03
*UCP2*	uncoupling protein 2 (mitochondrial, proton carrier)	352.9 ± 1.3	499.3 ± 1.4	41.5	6E-06	8E-04
*C1S*	complement component 1, s subcomponent	33.7 ± 1.4	47.5 ± 1.4	41.0	1E-04	6E-03
*PLEKHO2*	pleckstrin homology domain containing, family O member 2	138 ± 1.4	193.4 ± 1.3	40.1	8E-05	4E-03
*SNX10*	sorting nexin 10	148.6 ± 1.6	206.7 ± 1.5	39.1	5E-04	1E-02
*C1R*	complement component 1, r subcomponent	97.4 ± 1.4	134.2 ± 1.4	37.8	2E-03	3E-02
*SEMA3C*	sema domain, immunoglobulin domain (Ig), short basic domain, secreted, (semaphorin) 3C	176.5 ± 1.4	242.5 ± 1.4	37.4	1E-04	6E-03
*LOC100132793*	chromosome 8 open reading frame 88	12.1 ± 1.3	16.5 ± 1.3	36.2	8E-08	4E-05
*SIK1*	salt-inducible kinase 1	25.7 ± 1.4	35 ± 1.4	36.1	3E-05	2E-03
*LOC100133511*	uncharacterized LOC100131581	77 ± 1.4	104.6 ± 1.4	35.9	2E-04	7E-03
*MYL9*	myosin, light chain 9, regulatory	405.4 ± 1.3	549.5 ± 1.3	35.6	1E-05	1E-03
*EFEMP1*	EGF containing fibulin-like extracellular matrix protein 1	321 ± 1.4	434.9 ± 1.4	35.5	2E-03	3E-02
*GSTT2B*	glutathione S-transferase theta 2	21.3 ± 1.5	28.8 ± 1.4	35.3	8E-04	2E-02
*CXCR4*	chemokine (C-X-C motif) receptor 4	24.4 ± 1.6	32.9 ± 1.5	34.9	2E-03	4E-02
*GSTT2*	glutathione S-transferase theta 2	16.8 ± 1.5	22.7 ± 1.4	34.8	1E-03	2E-02
Upregulated genes						
*LOC100132761*	Uncharacterized	104.1 ± 2.1	44.3 ± 2.7	−57.4	3E-05	2E-03
*SLC7A10*	Solute carrier family 7 (neutral amino acid transporter light chain, asc system), member 10	655.1 ± 1.5	391.6 ± 1.9	−40.2	3E-03	4E-02
*PKD1L2*	polycystic kidney disease 1-like 2	203.3 ± 1.5	123.9 ± 1.7	−39.1	2E-03	3E-02
*LOC389342*	maltase-glucoamylase (alpha-glucosidase)	2047.4 ± 1.3	1253.2 ± 1.6	−38.8	2E-05	2E-03
*MYOC*	myocilin, trabecular meshwork inducible glucocorticoid response	126 ± 1.5	79.6 ± 1.6	−36.9	1E-03	2E-02
*C21orf81*	ankyrin repeat domain 20 family, member A11, pseudogene	98.1 ± 1.5	63.2 ± 1.6	−35.6	6E-04	1E-02
*LOC729222*	liprin-beta-1-like	49 ± 1.3	31.5 ± 1.3	−35.6	3E-11	5E-07
*COL8A1*	collagen, type VIII, alpha 1	456 ± 1.6	297.7 ± 1.5	−34.7	2E-04	7E-03
*SYT17*	synaptotagmin XVII	33 ± 1.5	21.6 ± 1.6	−34.4	4E-03	5E-02
*ANKRD20A1*	ankyrin repeat domain 20 family, member A4	417.4 ± 1.4	276.5 ± 1.4	−33.8	4E-06	6E-04
*LOC100128888*	uncharacterized	34.2 ± 1.3	22.8 ± 1.3	−33.4	1E-10	5E-07
*LOC654342*	lymphocyte-specific protein 1 pseudogene	55.1 ± 1.3	36.7 ± 1.4	−33.4	4E-07	1E-04
*PPFIBP1*	PTPRF interacting protein, binding protein 1 (liprin beta 1)	43.4 ± 1.3	29.1 ± 1.3	−32.9	7E-07	2E-04
*TSPAN18*	tetraspanin 18	162.5 ± 1.3	109.2 ± 1.3	−32.8	1E-08	1E-05
*NBPF20*	neuroblastoma breakpoint family, member 15	1226.5 ± 1.3	826.3 ± 1.5	−32.6	8E-05	4E-03
*FAM13A*	family with sequence similarity 13, member A	568.1 ± 1.4	395.2 ± 1.5	−30.4	3E-04	1E-02
*NFIC*	nuclear factor I/C (CCAAT-binding transcription factor)	308.9 ± 1.2	215.9 ± 1.3	−30.1	9E-11	5E-07
*FAM126B*	family with sequence similarity 126, member B	146.6 ± 1.3	103.2 ± 1.4	−29.6	7E-05	4E-03
*UGP2*	UDP-glucose pyrophosphorylase 2	32.6 ± 1.5	23 ± 1.5	−29.5	2E-04	7E-03
*MOGAT1*	monoacylglycerol O-acyltransferase 1	33.7 ± 1.4	23.9 ± 1.4	−29.2	3E-04	1E-02
*LOC100129905*	uncharacterized LOC100128905	38.2 ± 1.3	27 ± 1.4	−29.2	2E-05	2E-03
*FAM129A*	family with sequence similarity 129, member A	162.1 ± 1.4	115.1 ± 1.5	−29.0	5E-06	8E-04
*LOC100128899*	uncharacterized LOC100128979	172.3 ± 1.4	122.4 ± 1.4	−29.0	1E-03	2E-02
*GPT*	glutamic-pyruvate transaminase (alanine aminotransferase)	161.1 ± 1.3	114.6 ± 1.5	−28.9	2E-03	4E-02
*C1orf152*	profilin 1 pseudogene 2	30.1 ± 1.3	21.4 ± 1.3	−28.7	6E-06	8E-04

**Table 4:** Genes previously linked to PCOS, type 2 diabetes (T2D) and obesity in published GWAS with differential expression in adipose tissue from women with PCOS (n = 64) compared with controls (n = 30) (cohort 1).

Symbol	Illumina ID	Controls mean ± SD	PCOS mean ± SD	Difference (%)	*p*values	*Q*values
PCOS						
*RAB5B (Rabaptin5 β or member RAS oncogene family)*	2850626	1260.2 ± 1	1195.5 ± 1.1	–5.14	0.000	0.009
Diabetes						
*C2orf79 (peptidyl–tRNA hydrolase domain containing 1)*	3170725	64.3 ± 1.1	61.2 ± 1.1	–4.80	0.002	0.034
*CCDC102A (coiled–coil domain containing 102A)*	1660681	76.3 ± 1.2	70 ± 1.2	–8.21	0.000	0.009
*CLEC16A (C-type lectin domain family 16, member A)*	160224	134.4 ± 1.1	122.1 ± 1.1	–9.19	0.000	0.001
*IKZF4 (IKAROS family zinc finger 4 (Eos))*	5550608	29.6 ± 1.2	24.8 ± 1.2	–16.23	0.001	0.024
*IRS1 (Insulin receptor substrate)*	1710091	240.4 ± 1.3	257.3 ± 1.4	7.03	0.001	0.024
*MEG3 (maternally expressed 3 (non-protein coding))*	3360113	310.6 ± 1.3	235.4 ± 1.4	–24.22	0.001	0.021
*PHTF1 (putative homeodomain transcription factor 1)*	6900521	70.6 ± 1.1	79.6 ± 1.1	12.77	0.000	0.011
*POMC (proopiomelanocortin)*	5080072	34.6 ± 1.3	28.9 ± 1.3	–16.42	0.003	0.044
*PPARG (peroxisome proliferator–activated receptor gamma)*	830019	2866 ± 1.1	2478 ± 1.2	–13.54	0.001	0.025
*RBMS1 (RNA binding motif, single stranded interacting protein 1)*	4150639	526.1 ± 1.1	586.7 ± 1.1	11.52	0.000	0.009
*SGCG (8sarcoglycan. gamma (35kDa dystrophin-associated glycoprotein)*	5670082	138.5 ± 1.2	115.6 ± 1.2	–16.50	0.000	0.009
*SPRY2 (sprouty homolog 2 (Drosophila))*	6590575	284.4 ± 1.2	304.7 ± 1.2	7.12	0.000	0.010
*SVEP1 (sushi. von Willebrand factor type A, EGF and pentraxin domain containing 1)*	6180554	932.8 ± 1.2	837 ± 1.4	–10.27	0.000	0.001
*TNRC6A (trinucleotide repeat containing 6A)*	130386	19.5 ± 1.2	17.2 ± 1.2	–12.15	0.000	0.014
*TRAFD1 (TRAF-type zinc finger domain containing 1)*	1570129	90.4 ± 1.1	82.3 ± 1.1	–8.93	0.001	0.019
*TYK2 (tyrosine kinase 2)*	6900424	404.9 ± 1.1	380 ± 1.1	–6.17	0.003	0.045
*WWOX (WW domain containing oxidoreductase)*	6450189	34.5 ± 1.2	29 ± 1.2	–15.92	0.000	0.001
Obesity						
*ASAH1 (N-acylsphingosine amidohydrolase [acid ceramidase] 1)*	5420332	17.7 ± 1.2	19.9 ± 1.2	12.38	0.000	0.012
*LEPR (leptin receptor)*	6480348	25.7 ± 1.2	30.2 ± 1.2	17.63	0.002	0.030
*RTN4 (reticulon 4)*	780402	1202.6 ± 1.3	1468 ± 1.2	22.07	0.000	0.001
*RTN4 (reticulon 4)*	2230161	5505.2 ± 1.1	5988.2 ± 1.1	8.77	0.003	0.040
*TSEN34 (TSEN34 tRNA splicing endonuclease subunit)*	5890497	268.1 ± 1.1	282.9 ± 1.1	5.53	0.004	0.049

**Table 5:** Genes previously linked to type 2 diabetes and obesity in published GWAS with differential methylation (*Q* < 0.15) in adipose tissue from women with PCOS (n = 64) versus controls (n = 30) (cohort 1).

Symbol (name)	Illumina ID	Controls mean ± SD	PCOS mean ± SD	Difference (%)	*P*values	*Q*values
Diabetes						
*ARF5 (ADP-ribosylation factor 5)*	cg15802323	17.4 ± 1.4	19.1 ± 1.9	1.69	7.8E-05	0.15
*GALNTL4 (polypeptide N-acetylgalactosaminyltransferase 18)*	cg04807025	88.6 ± 0.7	87.9 ± 1.1	-0.78	7.1E-06	0.11
*R3HDML (R3H domain containing-like)*	cg17692403	15.3 ± 1.7	17.6 ± 2.4	2.26	9.2E-05	0.15
*PSMD6 (26S proteasome non-ATPase regulatory subunit 6)*	cg07662771	77.5 ± 2	74.7 ± 3.4	-2.78	1.2E-04	0.15
Obesity						
*PROX1 (prospero homeobox 1)*	cg22489498	9.2 ± 0.8	10 ± 0.8	0.79	1.5E-05	0.11
