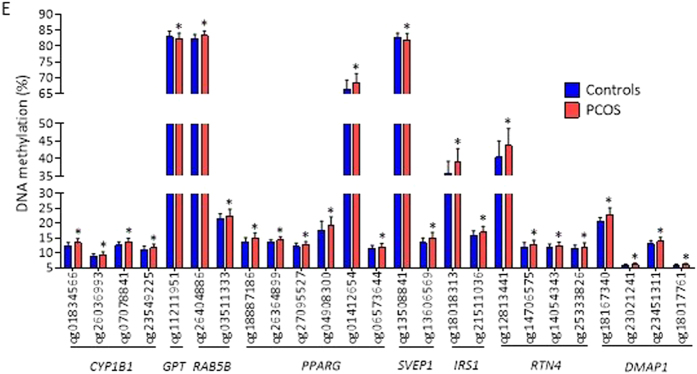# Erratum: Epigenetic and Transcriptional Alterations in Human Adipose Tissue of Polycystic Ovary Syndrome

**DOI:** 10.1038/srep25321

**Published:** 2016-05-09

**Authors:** Milana Kokosar, Anna Benrick, Alexander Perfilyev, Romina Fornes, Emma Nilsson, Manuel Maliqueo, Carl Johan Behre, Antonina Sazonova, Claes Ohlsson, Charlotte Ling, Elisabet Stener-Victorin

Scientific Reports 6: Article number: 22883;10.1038/srep22883 Published online: 03152016; Updated: 05092016

This Article contains errors.

In Table 3, the text in the first row ‘Down-regulated genes’ was incorrectly given as ‘Up-regulated genes’.

In addition, Fig. 2E was incorrectly labeled as Fig. 2ES. The correct Fig. 2E appears below as Fig. 1.

## Figures and Tables

**Figure 1:**