# Supplementary material for: Epigenetic and Transcriptional Alterations in Human Adipose Tissue of Polycystic Ovary Syndrome
Source: Sci Rep. 2016 Mar 15;6:22883. doi: 10.1038/srep22883 (PMC4791632; doi:10.1038/srep22883)
Supplement: Supplementary Information [file srep22883-s1.pdf]

Epigenetic and Transcriptional Alterations in Human Adipose Tissue of Polycystic Ovary Syndrome

Milana Kokosar[1], Anna Benrick[1], Alexander Perfilyev[2], Romina Fornes[3], Emma Nilsson[2] Manuel Maliqueo[3,4], Carl Johan Behre[5], Antonina Sazonova[6], Claes Ohlsson[7], Charlotte Ling[2] & Elisabet Stener-Victorin[4]*

[1] Department of Physiology, Institute of Neuroscience and Physiology, Sahlgrenska Academy, University of Gothenburg, Gothenburg, Sweden.

[2] Epigenetics and Diabetes, Department of Clinical Sciences, Lund University Diabetes Centre, Lund University, Clinical Research Centre, Malmö, Sweden.

[3] Laboratorio de Endocrinología y Metabolismo, Departamento de Medicina Occidente, Facultad de Medicina, Universidad de Chile, Santiago, Chile.

[4] Department of Physiology and Pharmacology, Karolinska Institutet, Stockholm, Sweden.

[5] The Wallenberg Laboratory and Sahlgrenska Center for Cardiovascular and Metabolic Research, Institute of Medicine, Sahlgrenska Academy, University of Gothenburg, Gothenburg, Sweden.

[6] Department of Obstetrics and Gynecology, Reproductive Medicine, Institute of Clinical Sciences, Sahlgrenska Academy, University of Gothenburg, Gothenburg, Sweden.

[7] Department of Internal Medicine, Centre for Bone and Arthritis Research, Institute of Medicine, Sahlgrenska Academy, University of Gothenburg, Gothenburg, Sweden.

Study participants. Women with PCOS and controls were recruited by advertising in local newspapers and in frequently visited places in the community. All women were ≥18 and ≤ 38 years of age. The eligibility criteria for women with PCOS were at least two of the following three signs; polycystic ovarian morphology (PCO), clinical signs of hyperandrogenism and oligo/amenorrhea. The criteria for ultrasound-verified PCO were ≥12 follicles 2–9 mm and/or ovarian volume ≥10 ml in one or both ovaries. A self-reported Ferriman-Gallwey (FG) score ≥8 was defined as hirsutism and acne was defined by a positive response to the question Do you have acne? Oligomenorrhea was defined as an intermenstrual interval >45 days and <8 menstrual bleedings in the past year. Amenorrhea was defined as <3 menstruations per year.

Exclusion criteria for controls were evidence of PCO morphology, excessive acne or hirsutism, or menstrual irregularities (cycles >35 days). Exclusion criteria for all women were any signs of other endocrine disorders such as hyperprolactinemia (s-prolactin <27µg/l), nonclassic congenital adrenal hyperplasia (17β hydroprogesterone <3nmol/l), and androgen-secreting tumors or autoimmune disorder, cancer, type I or type II diabetes or cardiovascular disease. Moreover, women had not received any pharmacological treatment within 12 weeks before inclusion, had not been pregnant, and had not been breastfeeding for 6 months before inclusion.

Supplementary Table Legends

Table S1: Genes differently expressed in adipose tissue from women with PCOS (n=64) and controls (n=30) ($Q < 0.05$) sorted in alphabetic order (cohort 1).

Table S2. Significant gene sets contributing to Z-score for expression pathways that are up-regulated in adipose tissue from women with PCOS versus controls (cohort 1).

Table S3. Significant gene sets contributing to Z-score for expression pathways that are down-regulated in adipose tissue from women with PCOS versus controls (cohort 1).

Table S4. Selected genes of relevance for PCOS found to be differentially expressed in cohort 1 were biological validated in cohort 2.

Table S5: Differential methylation in adipose tissue from women with PCOS and controls $P < 0.05$. (XLS)

Table S6: Differential methylation of CpG-sites in adipose tissue from women with PCOS and controls applying a $Q < 0.15$. (XLS)

Table S7: Significant gene sets with differential methylated genes in adipose tissue from women with PCOS versus controls. IPA analyses with $P < 0.05$. (XLS)

Table S8. Significant gene sets contributing to Z-score for differential methylated methylation pathways that are up-regulated in adipose tissue from women with PCOS versus controls (cohort 1).

Table S9. Significant gene sets contributing to Z-score for differential methylated methylation pathways that are down-regulated in adipose tissue from women with PCOS versus controls (cohort 1).

Table S10: Differential methylation in adipose tissue from women with PCOS and controls based on the cross reactive probes $P < 0.05$. (XLS)

Table S11: Differential DNA methylation in adipose tissue ($Q < 0.15$ and the range of difference in β-value is 0.3 - 3%) concurrent with an inverse change in mRNA expression ($P < 0.05$) of the nearest gene between PCOS women and controls (cohort 1).

Table S12: Differential methylation of DNA methyltransferases (*DNMTs*), a family of enzymes catalyzing the transfer of a methyl group to DNA, in adipose tissue from women with PCOS and controls.

Table S13. Spearman correlations with correction for multiple testing ($Q < 0.05$) between methylation and expression for significantly differentially expressed genes and CPG sites within the cis distance 500 kb upstream and 100 kb downstream of the gene.
